# Supplementary material for: Identifying Probable Dementia in Undiagnosed Black and White Americans Using Machine Learning in Veterans Health Administration Electronic Health Records
Source: Big Data Cogn Comput. Author manuscript; Available in PMC 2025 Sep 17. (PMC12440470; doi:10.3390/bdcc7040167)
Supplement: Table S1a and S1b [file NIHMS2100280-supplement-Table_S1a_and_S1b.pdf]

Supplemental Table 1a. Top 20 most important variables in the support vector machine (SVM) model for Black American (BA) Veterans, ranked by absolute value of variable weight in the model.

Type	***Variable Definition***	***Weight***
Topic	memory, cognitive, dementia, impairment, wife, loss, problems, donepezil, testing, mild, ...	0.454
Demographic	Age (normalized to be between 0 and 1 with 0=65yrs and 1=85yrs)	0.237
Diagnosis	Memory loss/anemia (ICD9: 780.93; ICD10: R41.[1-3])	0.144
Topic	weight, move, program, management, obesity, health, loss, bmi, risks, discussed, ...	**- 0.116**
Procedure	CT HEAD/BRAIN, W/O DYE (CPT: 70450)	0.103
Topic	wife, home, reports, states, accompanied, spoke, husband, concerned, house, giving, ...	0.102
Topic	time, correct, point, work, back, score, points, state, clock, year, ...	0.092
Topic	point, assistance, points, independently, score, total, shopping, batching, activities, ability, ...	0.090
Topic	mri, brain, ct, head, acute, small, left, matter, intracranial, white, ...	0.082
Topic	daughter, mh, home, father, spoke, family, br, states, lives, stated, ...	0.082
Topic	nursing, rehab, home, placement, facility, care, va, nh, cnh, contract, ...	0.071
Diagnosis	Mild cognitive impairment (ICD9: 331.83; ICD10: G31.84)	0.070
Procedure	VITAMIN B-12 (CPT: 82607)	0.066
Diagnosis	Psychotic disorder with hallucinations (ICD9: 293.82; ICD10: 294.[8,9], F06.[0,8])	0.063
Topic	cva, stroke, weakness, sided, left, cerebralvascular, hemiparesis, residual, speech, accident, ...	0.062
Topic	wife, mrs, spouse, spoke, called, call, husband, stated, mr, home, ...	0.062
Topic	walker, cane, gait, walking, ambulation, walk, rollator, ambulates, falls, difficulty, ...	0.058
Topic	tremor, parkinson, tremors, disease, hand, gait, mild, sinemet, pd, levodopa	0.057
Topic	medication, pill, box, medications, taking, med, pills, meds, bottle, bottles, ...	0.057
Procedure	SYPHILIS TEST NON-PREP QUAL (CPT: 86592)	0.056

Supplemental Table 1b. Top 20 most important variables in the support vector machine (SVM) model for White American (WA) Veterans, ranked by absolute value of variable weight in the model.

Type	***Variable Definition***	***Weight***
Topic	memory, cognitive, dementia, impairment, wife, loss, problems, donepezil, testing, mild, ...	0.468
Demographic	Age (normalized to be between 0 and 1 with 0=65yrs and 1=85yrs)	0.225
Diagnosis	Memory loss/anemia (ICD9: 780.93; ICD10: R41.[1-3])	0.180
Topic	wife, home, reports, states, accompanied, spoke, husband, concerned, house, giving, ...	0.126
Topic	weight, move, program, management, obesity, health, loss, bmi, risks, discussed, ...	**- 0.120**
Topic	point, assistance, points, independently, score, total, shopping, batching, activities, ability, ...	0.117
Topic	mri, brain, ct, head, acute, small, left, matter, intracranial, white, ...	0.102
Topic	time, correct, point, work, back, score, points, state, clock, year, ...	0.100
Topic	tremor, parkinson, tremors, disease, hand, gait, mild, sinemet, pd, levodopa, ...	0.087
Procedure	CT HEAD/BRAIN, W/O DYE (CPT: 70450)	0.082
Topic	daughter, mh, home, father, spoke, family, br, states, lives, stated, ...	0.079
Diagnosis	Psychotic disorder with hallucinations (ICD9: 293.82, 294.[8,9]; ICD10: F06.[0,8])	0.072
Topic	nursing, rehab, home, placement, facility, care, va, nh, cnh, contract, ...	0.069
Topic	walker, cane, gait, walking, ambulation, walk, rollator, ambulates, falls, difficulty, ...	0.067
Diagnosis	Mild cognitive impairment (ICD9: 331.83; ICD10: G31.84)	0.065
Topic	memory, average, cognitive, test, range, impaired, functioning, performance, evaluation, testing, ...	0.064
Diagnosis	Parkinson's disease (ICD9: 332.0; ICD10: G20., G21.4)	0.062
Topic	injury, head, fall, fell, hit, back, ago, fracture, rib, trauma, ...	0.058
Procedure	SYPHILIS TEST NON-PREP QUAL (CPT: 86592)	0.057
Procedure	VITAMIN B-12 (CPT: 82607)	0.055
